# Telehealth follow‐up consultations for melanoma patients during the COVID‐19 pandemic: Patient and clinician satisfaction

**DOI:** 10.1002/cam4.6679

**Published:** 2023-11-06

**Authors:** Ali Al‐Rikaby, Ahmad Sulaiman, Jake R. Thompson, Robyn P. M. Saw, Frances Boyle, Nicole Taylor, Matteo S. Carlino, Rachael L. Morton, Omgo E. Nieweg, John F. Thompson, Iris Bartula

**Affiliations:** ^1^ Faculty of Medicine and Health The University of Sydney Sydney New South Wales Australia; ^2^ Melanoma Institute Australia The University of Sydney North Sydney New South Wales Australia; ^3^ Department of Melanoma and Surgical Oncology Royal Prince Alfred Hospital Sydney New South Wales Australia; ^4^ Patricia Ritchie Centre for Cancer Care and Research Mater Hospital North Sydney New South Wales Australia; ^5^ Department of Medical Oncology Westmead and Blacktown Hospitals Sydney New South Wales Australia; ^6^ NHMRC Clinical Trials Centre, Faculty of Medicine and Health The University of Sydney Camperdown, Sydney New South Wales Australia

**Keywords:** COVID‐19, melanoma, satisfaction, supportive care, telehealth

## Abstract

**Introduction:**

The COVID‐19 pandemic caused rapid implementation of telehealth for melanoma follow‐up care in Australia. This study explores Australian melanoma patients and clinicians' level of satisfaction with telehealth.

**Methods:**

A cross‐sectional study was conducted across three specialist melanoma centres in Sydney, Australia. Melanoma patients (all stages) and clinicians completed mixed methods surveys seeking socio‐demographic and clinical information and questionnaires to assess satisfaction with telehealth. Additionally, patients completed measures of quality of life, fear of cancer recurrence and trust in their oncologist. Patients and clinicians provided open‐ended responses to qualitative questions about their perceptions of telehealth.

**Results:**

One hundred and fifteen patients and 13 clinicians responded to surveys. Telephone was used by 109 (95%) patients and 11 (85%) clinicians. Fifty‐seven (50%) patients and nine (69%) clinicians preferred face‐to‐face consultations, 38 (33%) patients and 3 (23%) clinicians preferred a combination of face‐to‐face and telehealth consultations. Five (4%) patients and nil clinicians preferred telehealth consultations. Patients diagnosed with early‐stage melanoma, using telehealth for the first time, who have lower trust in their oncologist, and having higher care delivery, communication and supportive care concerns were likely to report lower satisfaction with telehealth. Open‐ended responses were consistent between patients and clinicians, who reported safety, convenience and improved access to care as major benefits, while identifying personal, interpersonal, clinical and system‐related disadvantages.

**Discussion:**

While telehealth has been widely implemented during COVID‐19, the benefits identified by patients and clinicians may extend past the pandemic. Telehealth may be considered for use in conjunction with face‐to‐face consultations to provide melanoma follow‐up care.

## 1 INTRODUCTION

Australia has the highest melanoma incidence rate in the world.^1^ Within 2 years of initial diagnosis 13.4% of patients develop recurrence of the disease,^2^ and regular follow‐up is part of standard melanoma care. For some higher risk patients, follow‐up appointments are recommended as frequently as every 3 months.^3^ These consultations can also benefit patients' emotional well‐being, as they can be reassuring and help manage anxiety, fear of cancer recurrence and promote preventative behaviours such as skin self‐examinations.^4^

The COVID‐19 health crisis led to implementation of lockdown measures to prevent the spread of the virus in Australia.^5^ With government travel restrictions in place, and widespread fear of COVID‐19 infection, healthcare clinics restricted their face‐to‐face services to essential interactions. In response, the Australian Government introduced temporary Medicare subsidies for healthcare practitioners to offer telehealth when appropriate.^6^ This led to increased use of telehealth to deliver melanoma follow‐up care.

Telehealth, or telemedicine, is a method of delivering clinical consultations remotely using telephones or video links, bridging the ‘geographical gap’ between patients and clinicians while offering an accessible alternative to face‐to‐face consultations.^7^ A primary benefit of telehealth is increasing patient access to healthcare regardless of location, travel restrictions and availability of face‐to‐face services. As there are also downsides to telehealth (e.g. inability to conduct physical examination, express empathy as easily, particularly when there is bad news and patient is distressed), there is a need to assess the satisfaction with this mode of service delivery. While patient and clinician satisfaction with telehealth has been described for other cancer types,^8^, ^9^, ^10^, ^11^, ^12^, ^13^, ^14^, ^15^, ^16^, ^17^, ^18^, ^19^, ^20^, ^21^, ^22^, ^23^ little is known about satisfaction with telehealth for follow‐up of melanoma patients. In melanoma context, where visual and physical examinations are a key feature of follow‐up consultations, telehealth may be perceived differently compared to other cancers. Therefore, this study aims to explore the satisfaction of melanoma patients and clinicians with telehealth consultations conducted during the COVID‐19 pandemic, and to identify related demographic, clinical and psychological factors associated with patient satisfaction.

## 2 METHODOLOGY

### 2.1 Study design

This was a prospective, cross‐sectional study which utilised self‐reported questionnaires and captured both quantitative and qualitative data. Ethical approval was granted by Sydney Local Health District—Royal Prince Alfred Zone (2020/ETH02519). Participants provided written informed consent.

### 2.2 Setting and participants

The study recruited melanoma patients and clinicians from surgical clinics at the Poche Centre facility of Melanoma Institute Australia (MIA), the Department of Melanoma and Surgical Oncology at Royal Prince Alfred Hospital and the Medical Oncology Unit at the Crown Princess Mary Cancer Centre, Westmead Hospital, all located in New South Wales, Australia. Patients eligible for this study were identified by the clinic sites and sent a study invitation package via mail. They could complete the survey online using a link provided in the package or request a paper survey by returning an expression‐of‐interest form. Melanoma clinicians who were eligible for this study were identified and invited via email to complete the survey electronically (via an embedded link) by the administrative staff of the MIA Co‐Medical Directors.

Patients were eligible for the study if they met the following criteria: (1) a previous diagnosis of melanoma (any American Joint Committee on Cancer 8th Edition^24^ stage of disease; for the current study, patients were classified to have either early‐stage disease, defined as Stage 0, I or II, or advanced stage, defined as Stage III or IV); (2) received at least one telehealth consultation from a MIA‐affiliated clinician at one of the participating study sites between March 2020 and February 2022; (3) had previously consented to be included in MIA's Melanoma Research Database; (4) had English language skills permitting them to understand the study materials and the capacity to provide informed consent; and (5) were ≥18 years of age. Patients were excluded if they were diagnosed with any other skin condition, including non‐melanoma skin cancer and if the telehealth consultations they participated in were not related to their melanoma management.

Clinicians were eligible for the study if they (1) practiced as a healthcare professional providing care, support and management to people diagnosed with melanoma; (2) provided these services at one of the participating study sites; (3) provided at least one telehealth consultation for melanoma management between March 2020 and February 2022. Clinicians were excluded if they only used telehealth to provide management of issues other than melanoma.

### 2.3 Patient quantitative outcomes

Patient surveys were available either in paper form or electronically (depending on patient preference). Demographic questions collected information on patient characteristics such as age, sex, living arrangements, language spoken at home, health insurance status, education level and income. Patients were asked about the postcode of their primary residence, which was classified into metropolitan, rural and remote according to the Accessibility/Remoteness Index of Australia (ARIA), a nationally recognised classification system. Clinical questions regarding diagnosis and treatment of melanoma were also included.

Patient satisfaction was evaluated using the Telemedicine Satisfaction and Usefulness Questionnaire (TSUQ),^25^ a 21‐item Likert‐style survey with acceptable psychometrics, where higher scores indicate higher satisfaction. No cut‐off score indicating satisfaction with telehealth exists in the literature, therefore scores of 4/5 (agree) and 5/5 (strongly agree) were used to indicate satisfaction with telehealth. An additional four questions regarding the satisfaction with the telehealth sessions were taken from a previous research study.^26^

Patient trust in their oncologist was measured using the Trust in Oncologist Scale—Short Form (TiOS‐SF).^27^ This 5‐item short form has acceptable psychometrics, and measures perceived competence, honesty, fidelity, caring and general trust in medical practitioners. It is answered using a five‐point Likert‐scale. A total score combines item scores, ranges from 5 to 25, with higher scores indicating higher levels of trust.^27^, ^28^

Patient quality of life was measured using two surveys. The European Organization for the Research and Treatment of Cancer Core Quality of Life Questionnaire Version 3 (QLQ‐C30)^29^ which consists of six single‐item scales (dyspnoea, insomnia, appetite loss, constipation, diarrhoea and financial difficulties), three multi‐item symptom scales (fatigue, nausea and vomiting, and pain), five multi‐item functional scales (physical, role, emotional, cognitive and social) and a multi‐item global health status/quality of life scale, responded to using Likert‐type scales. The QLQ‐C30 has acceptable psychometric properties, with scores ranging from 0 to 100 and higher scores indicating better functioning and worse symptomology on functional and symptom scales, respectively.^29^, ^30^ The Melanoma Concerns Questionnaire (MCQ‐28)^31^ examines quality of life across four multi‐item domains (disease prognosis and acceptance, treatment concerns and future disease risk, supportive care and care delivery and communication). The MCQ‐28's response options are consistent with the QLQ‐C30, with scores ranging from 0 to 100. It has acceptable psychometric properties and follows the same response structure as the QLQ‐C30, except for the treatment concerns and future disease risk domains, where higher scores indicate increased concerns.^31^

Fear of cancer recurrence was measured using the Fear of Cancer Recurrence Inventory 9‐item Short Form (FCRI‐9).^32^ The FCRI‐9 measures fear of cancer recurrence severity using a five‐point Likert‐type scale. Item scores are summed to produce a total score (range 0–36), where higher scores indicate higher fear of cancer recurrence severity. The FCRI‐9 has acceptable psychometric properties.^33^

### 2.4 Clinician quantitative outcomes

Clinician surveys were available electronically. Demographic questions collected information on clinician characteristics such as age, sex, career stage and years working in a melanoma‐related role.

Clinician satisfaction with telehealth was measured using the Health Optimum telemedicine acceptance questionnaire (HOTAQ),^34^ a survey consisting of eight items measuring clinicians' perceptions of telehealth. Although the HOTAQ has acceptable psychometric properties,^34^ due to differing response options across questions, the items are presented individually, rather than in an aggregate format. Clinicians were also asked an additional 11 questions from a previous research study.^26^

Medicare Benefits Schedule (MBS) includes wide range of healthcare consultations, procedures and tests that are funded by Australian Government. Healthcare services contained in the MBS are allocated unique number (MBS Item), which contains the service description and the Schedule Fee that is funded through Medicare. When new services are funded by the Government, such as telehealth consultations during COVID‐19, new MBS items are created. As part of the survey, clinicians were also asked which MBS items were usually selected when providing face‐to‐face or telehealth consultations and asked to comment on the suitability of these items for melanoma care. These responses were open‐ended.

### 2.5 Qualitative outcomes

Qualitative questions for both patients and clinicians aimed to elicit factors that influenced their perception of telehealth, benefits and barriers regarding adoption, and preferences for future consultation. These questions were ‘*What factors influenced your satisfaction with telehealth? Why?*’, ‘*What are the most important benefits of telehealth for you? Why?*’, ‘*What are the biggest barriers of telehealth for you? Why?*’, ‘*What could be done to make telehealth more helpful?*’ and ‘*Do you prefer telehealth or face‐to‐face appointments? Why?*’

### 2.6 Data analysis

Descriptive statistics, including means, standard deviations (SD), frequency counts and proportions were used to report patient and clinician demographics. To investigate bivariate associations between clinical and demographic variables and overall TSUQ score, parametric tests were used if assumptions (no outliers, normal distribution, equal variances and homoscedasticity) were met. Parametric tests included: (1) Pearson correlation coefficient (to test associations between TSUQ and a continuous variables), (2) independent samples *t*‐test (to test differences in TSUQ scores between two groups) and (3) Analysis of Variance (to test differences in TSUQ scores between three independent groups). If the assumptions were violated, non‐parametric alternatives to these tests were used: (1) Spearman's rank correlation, (2) Mann–Whitney *U* and (3) Kruskal–Wallis test. Effect sizes were reported using Cohen's *d* (*d*; 0.2–small, 0.5–medium, 0.8–large) and *η* ^2^ (*η* ^2^; 0.01–small, 0.06–medium, 0.138–large) for independent samples *t*‐tests and analysis of variance tests, respectively. A two‐sided *p* < 0.05 indicated statistical significance. Quantitative analyses were conducted using SPSS (v.26, 2019).

Patient and clinician responses to qualitative questions were analysed using a standard thematic analysis procedure^35^ to identify factors that may be related to perception of telehealth. IB and AA conducted thematic analysis, with no a priori hypotheses about themes that might emerge. First, both authors familiarised themselves with all the data by reading and re‐reading the responses to qualitative questions, which allowed them to develop initial codes. These codes were then combined to form initial subthemes and themes, which were then reviewed for consistency. Iterative analysis was used, where analysis moved from more specific to general (i.e. codes to sub‐themes to themes), with the aim to arrive at increasingly broader categories. This process was followed separately for patients and clinicians, with the comparison between sub‐themes and themes occurring only once the final themes for each sample were established. NVivo (2020) was used to organise qualitative data.

## 3 RESULTS

### 3.1 Patient quantitative results

Three hundred and sixty‐two patients were invited to participate in the study. The survey was completed by 115 melanoma patients (32% response rate). Table 1 reports the clinical and socio‐demographic features of respondents. The telephone was used in 109 (95%) consultations.

**TABLE 1:** Patient and clinician demographics.

Patient demographics (*n* = 115)	
Sex (*n*, %)	
Males	63 (55%)
Females	52 (45%)
Age in years (mean, SD)	68 (13)
Residence (*n*, %)	
Metropolitan	70 (61%)
Regional/remote	42 (37%)
Missing	3 (2%)
Living arrangement (*n*, %)	
Living alone	15 (13%)
Living with someone	100 (87%)
Employment status (*n*, %)	
Retired	77 (67%)
Employed	31 (27%)
Unemployed	3 (3%)
Missing	4 (3%)
Highest educational attainment (*n*, %)	
Secondary	52 (45%)
Tertiary	38 (33%)
Vocational	23 (20%)
Missing	2 (2%)
Annual income, before tax (*n*, %)	
<$62,000 AUD	73 (64%)
$62,000–$121,999 AUD	12 (10%)
≥$122,000 AUD	7 (6%)
Missing	23 (20%)
Time since diagnosis (*n*, %)	
<12 months	16 (14%)
13–23 months	15 (13%)
≥24 months	80 (70%)
Missing	4 (3%)
Melanoma stage (*n*, %)	
I	34 (30%)
II	18 (16%)
III	23 (20%)
IV	30 (26%)
Unknown	10 (9%)
Current melanoma management (*n*, %)^a^	
Routine follow‐up	67 (58%)
Immunotherapy	12 (10%)
Targeted therapy	8 (7%)
No current treatment	8 (7%)
Radiation therapy	4 (4%)
Other treatments	10 (17%)
Most recent telehealth session (*n*, %)	
2020	46 (40%)
2021	59 (51%)
2022	6 (5%)
Missing	4 (3%)
Telehealth session type (*n*, %)	
Telephone	109 (95%)
Videoconference	3 (3%)
Missing	3 (3%)

Clinician demographics (*n* = 13)			
Sex (*n*, %)			
Males		7 (54%)	
Females		6 (46%)	
Age in years (mean, SD)		53 (11)	
Profession (*n*, %)			
General practitioner		4 (31%)	
Medical oncologist		3 (23%)	
Dermatologist		2 (15%)	
Surgical oncologist		2 (15%)	
Radiation oncologist		1 (8%)	
Melanoma nurse		1 (8%)	
Career stage (*n*, %)			
Early (<10 years)		1 (8%)	
Mid (10–20 years)		4 (31%)	
Late (>20 years)		8 (62%)	
Years working in melanoma (*n*, %)			
0–10 years		3 (23%)	
11–20 years		6 (46%)	
21–30 years		3 (23%)	
>30 years		1 (8%)	
Telehealth session type (*n*, %)			
Telephone		11 (85%)	
Videoconference		2 (15%)	
Years using telehealth (*n*, %)			
<2 years		12 (92%)	
≥2 years		1 (8%)	
Telehealth frequency			
≥1 time a week		9 (69%)	
<1 time a week		2 (15%)	
Missing		2 (15%)	
MBS item numbers used^a^			
**Face‐to‐face**		**Telehealth**	
MBS item	(*n*, %)	MBS item	(*n*, %)
105	5 (38%)	91822	3 (23%)
104	4 (31%)	91823	3 (23%)
116	3 (23%)	91825	2 (15%)
110	2 (15%)	91893	2 (15%)
132	2 (15%)	91810	2 (15%)
53	2 (15%)	91812	1 (8%)
133	1 (8%)	91824	1 (8%)
36	1 (8%)	91835	1 (8%)
Nil	1 (8%)	Nil	1 (8%)

Abbreviations: MBS, Australian medicare benefits schedule; SD, standard deviation.

^a^ Not mutually exclusive.

**FIGURE 1:**
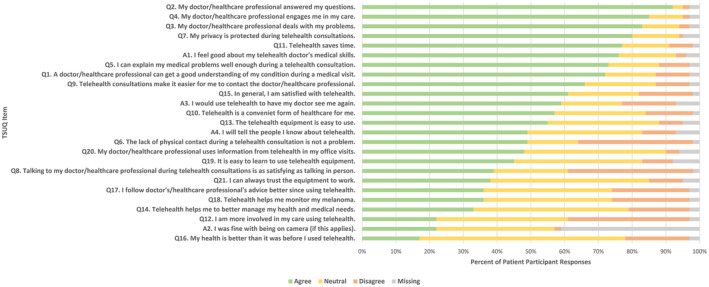
Patient responses to the TSUQ and additional questions. TSUQ, Telemedicine Satisfaction and Usefulness Questionnaire.

The responses to the telehealth satisfaction (TSUQ, additional) items are reported in Figure 1. Most patients agreed that their doctor answered their questions (*n* = 107, 92%), engaged them in their care (*n* = 98, 85%) and dealt with their problems (*n* = 94, 83%). Most patients thought their privacy was protected during telehealth (*n* = 92, 80%) and that telehealth saved time (*n* = 89, 77%). Overall, 88 (76%) patients felt good about their doctor's skills. In contrast, the least positively rated items saw only a minority of patients agree that telehealth improved their health (*n* = 19, 17%), involvement in their care (*n* = 25, 22%) and management of their medical needs (*n* = 37, 33%), while 41 (36%) patients agreed that telehealth improved monitoring of their melanoma.

Patients with early‐stage melanoma reported lower satisfaction with telehealth than patients with advanced melanoma (*t*(87) = −2.88, *p* < 0.01, *d* = −0.61). Patients who used telehealth for the first time had significantly lower satisfaction than patients who had used it before (*F*(2,94) = 6.12, *p* < 0.01, *η* ^2^ = 0.13). Additionally, significant weak correlations were found between higher satisfaction with telehealth and higher trust in the oncologist (*r*(90) = 0.28, *p* < 0.01), lower care delivery and communication concerns (*r*(91) = −0.29, *p* < 0.01) and fewer supportive care concerns (*r*(90) = 0.29, *p* < 0.01). Age, sex, rural status, living arrangements, employment status, educational attainment, annual income, time since diagnosis, clinical trial participation, overall quality of life, fear of cancer recurrence, disease prognosis and acceptance and treatment concerns and future disease risk were not associated with patient satisfaction with telehealth (*p* > 0.05; see Table S1).

### 3.2 Clinician quantitative results

Out of 50 melanoma clinicians invited, the survey was completed by 13 (26% response rate) from six different categories of practice (Table 1). Over half (*n* = 8, 62%) of the clinicians described themselves as being in the later stage of their careers (i.e. >20 years in practice), with 10 (77%) working over 10 years in melanoma. The majority of clinicians conducted telehealth consultations via telephone (*n* = 11, 85%), within the past 2 years (*n* = 12, 92%). A majority reported they used telehealth to conduct consultations at least once a week (*n* = 9, 69%).

**FIGURE 2:**
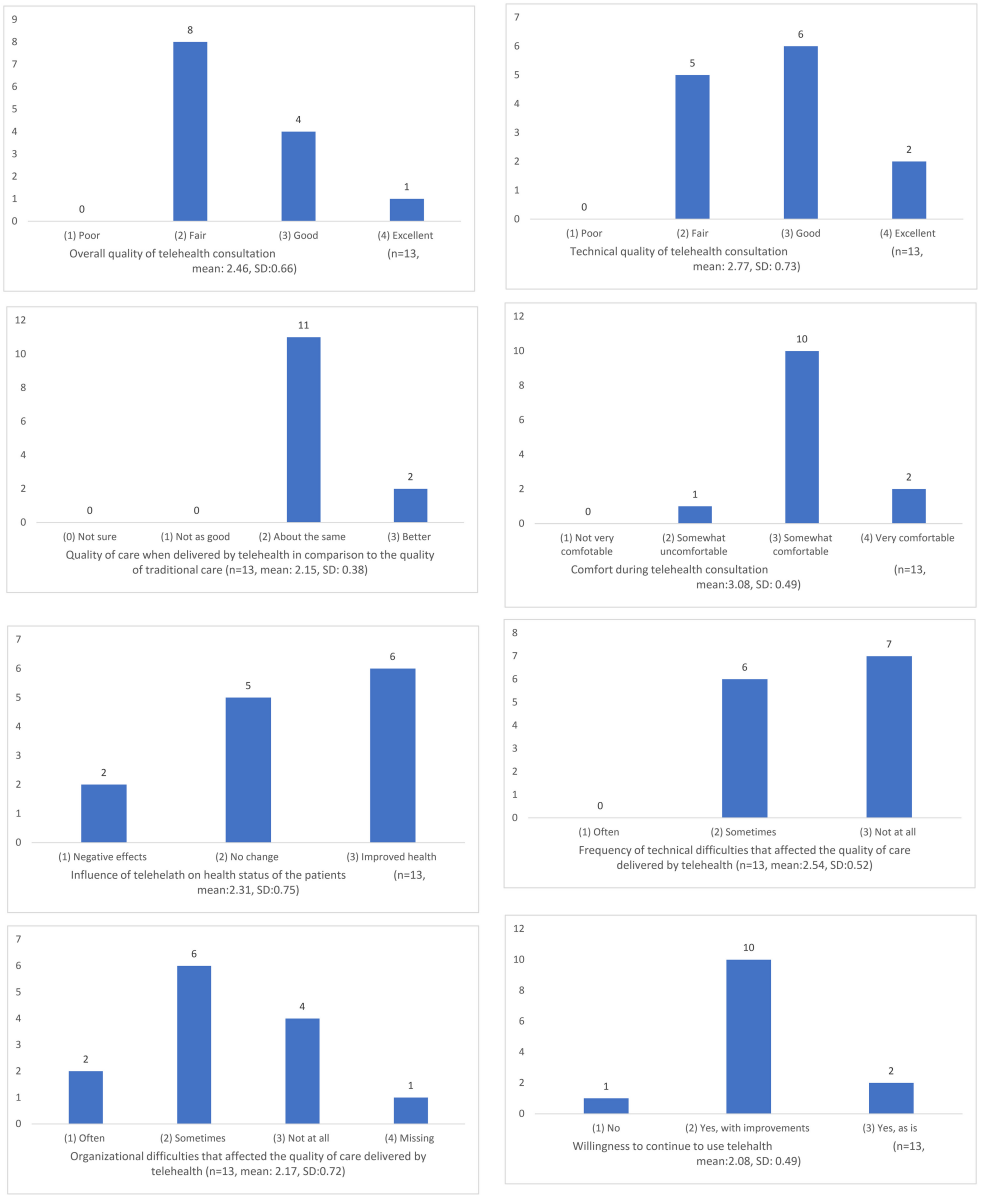
Frequency of clinician responses on the Health Optimum telemedicine acceptance questionnaire (HOTAQ).

HOTAQ scores (Figure 2) indicated most melanoma clinicians perceived telehealth consultations to be of fair overall (*n* = 8, 62%) and good technical quality (*n* = 6, 46%), with some logistic difficulties (*n* = 6, 46%) reported. Eleven (85%) thought that the quality of telehealth consultation was similar to that of face‐to‐face consultations, while 10 (77%) felt somewhat comfortable when delivering telehealth consultations. Six (46%) clinicians thought that telehealth improved the health of their patients and 10 (77%) would consider using it again, but with some improvements.

**FIGURE 3:**
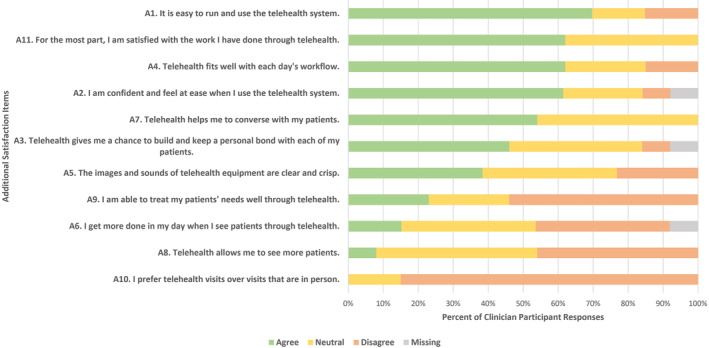
Clinician responses to the additional satisfaction questions.

Figure 3 outlines the percentages of responses to the additional satisfaction items. Clinicians reported that telehealth was easy to use (*n* = 8, 69%), they were satisfied with the work they have done through telehealth (*n* = 8, 61%), they experienced telehealth fitting well with their workload (*n* = 8, 61%) and they felt confident in using telehealth (*n* = 8, 61%). The most negatively rated items regarded the preference for telehealth over face‐to‐face consultations (*n* = 0, 0%), telehealth allowing clinicians to see more patients (*n* = 1, 8%) and increasing daily productivity (*n* = 2, 16%).

#### 3.2.1 Clinician opinions about medicare benefits schedule items

The most frequently used MBS items were 105 (specialist follow‐up consultation), 104 (specialist initial consultation) and 116 (physician follow‐up consultation) for face‐to‐face consultations, and 91822, 91823 and 91825 for their respective telehealth equivalents. Clinicians thought that the number of sessions per patient is ‘*…fair, given it is equivalent of [sic] in person number*’ (*Medical oncologist*); however, the ‘*Rebate is too low. [Telehealth] Item number doesn't reflect the complex nature of some of the consultations*’. (*General practitioner*).

#### 3.2.2 Patient and clinician preference for telehealth

Fifty‐seven (50%) patients and nine (69%) clinicians preferred face‐to‐face consultations, with an additional 38 (33%) patients and 3 (23%) clinicians preferring a combination of face‐to‐face and telehealth consultations. Five (4%) patients and nil clinicians preferred telehealth consultations, with 15 (13%) patients and 1 clinician (8%) reporting no preference or no response.

### 3.3 Patient and clinician qualitative responses

#### 3.3.1 Disadvantages of telehealth

**FIGURE 4:**
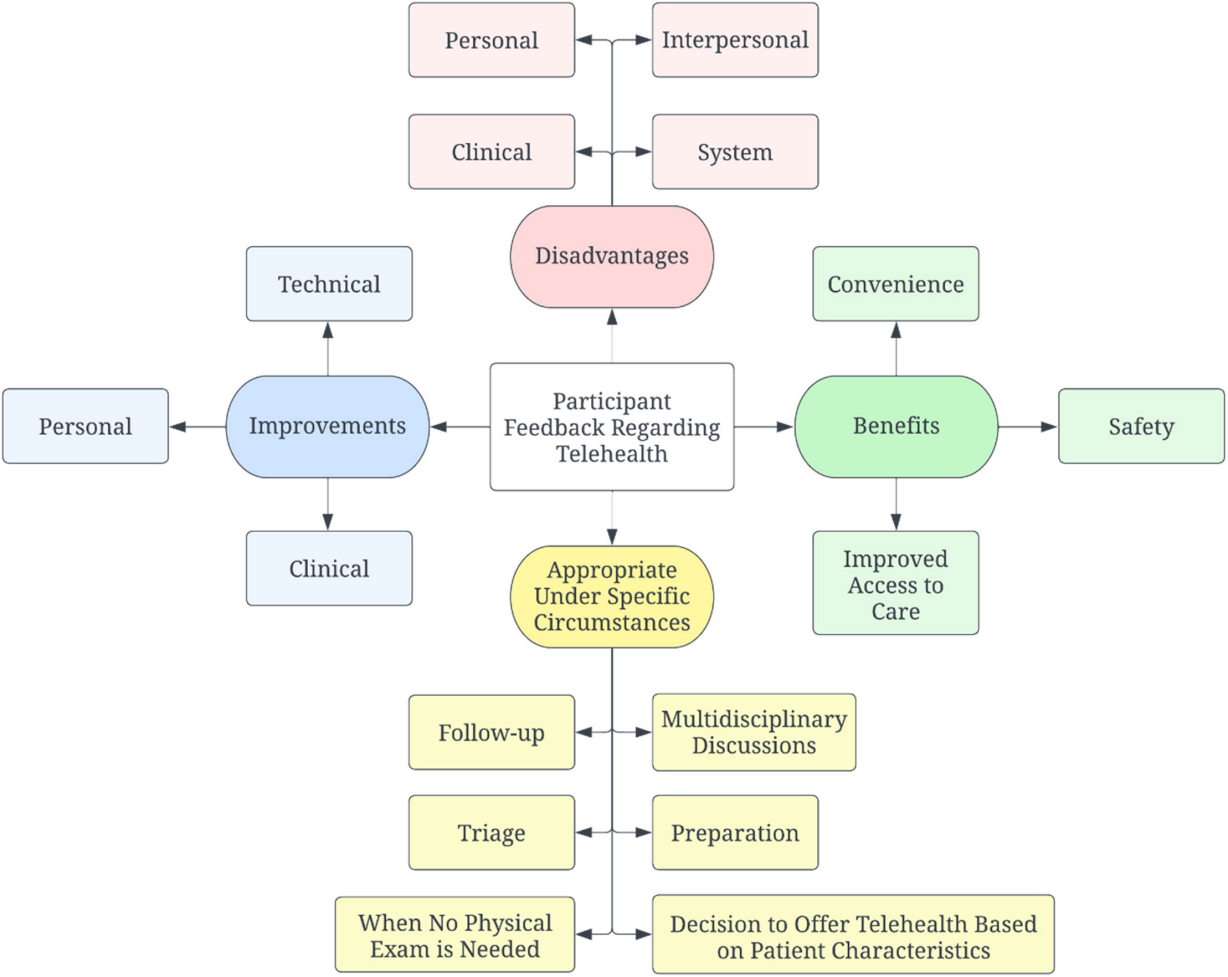
Mind map of identified themes from participant qualitative data.

Both patients and clinicians identified telehealth disadvantages at clinical, personal, interpersonal and system levels (Table 2; Figure 4). They agreed that a major clinical shortcoming of telehealth was the inability to conduct physical examinations. At a personal level, patients and clinicians identified unfamiliarity and discomfort with telehealth and not having access to the right equipment, as disadvantages. Furthermore, some patients found telehealth distracting at times. At the interpersonal level, patients and clinicians agreed that when using telehealth, communication can be challenging, with rapport difficult to establish and maintain, at times leading to increased fatigue for some clinicians. At the system level, patients and clinicians agreed that some administrative difficulties existed, while patients also acknowledged that technological shortcomings sometimes interfered with their telehealth experience.

**TABLE 2:** Qualitative themes and responses from participants.

Theme	Sub‐theme	Patient responses	Clinician responses
*Disadvantages of telehealth*			
Clinical	Telehealth is not adequate for physical exams	‘*Blood pressure cannot be taken, weight, hands on feeling lymph nodes,* etc. *[in] groin area*’. Male, 86, early‐stage melanoma	‘*Melanoma patients requiring skin checks and follow‐up is a very physical process. Telehealth is obviously not able to perform this task*’. General practitioner
Personal	Not familiar/comfortable with telehealth	‘*…having difficulty understanding all new technology*’. Male, 77, advanced stage melanoma	‘*I only use phone as the video function is difficult for patients (sometimes me) to use*’. Radiation oncologist
	Not having the right equipment	‘*I did not have the technology to do the video call, so I had to do it over the phone*’. Male, 57, early‐stage melanoma	‘*Logistics of using it ‐ patients may not have access to sufficient technology to provide useful interaction*’. Surgical oncologist
	Telehealth can be distracting	‘*… easily distracted when on the phone*’. Female, 37, advanced stage melanoma	*Sub‐theme did not emerge in clinician sample*
Interpersonal	Communication is difficult during telehealth	‘*I find it may not convey the emotion of a patient to a health professional…*’ Female, 56, advanced stage melanoma	‘*…cannot pick up on non‐verbal clues on telephone*’. Medical oncologist
	Rapport is difficult to establish and maintain	‘*…feel disconnected from doctor*’. Male, 57, early‐stage melanoma	‘*Less interaction/rapport/connection with patients…*’ Medical oncologist
	Telehealth can be exhausting	*Sub‐theme did not emerge in patient sample*	‘*It's exhausting and you have to work harder as body language is different*’. Medical Oncologist
Health system	Administrative difficulties	‘*…there has been confusion with appointment times*’. Male, 79, advanced stage melanoma	‘*Documentation issues. Inadequate electronic record systems*’. General practitioner
	Technological shortcomings	‘*Communications is not the best where I live*’. Female, 67, unknown stage	*Sub‐theme did not emerge in clinician sample*
*Benefits of telehealth*			
Convenience	Time saving	‘*Time saving ‐ usually a full day at clinic. No waiting to be seen*’. Female, 74, advanced stage melanoma	*Sub‐theme did not emerge in clinician sample*
	Cost saving	‘*…saving on fuel costs and the trauma negotiating city traffic*’. Male, 70, advanced stage melanoma	*Sub‐theme did not emerge in clinician sample*
	Overcoming geographical distance	‘*I live in rural NSW. Travelling to the city, accommodation and time off work were required prior to telehealth*’. Female, 43, early‐stage melanoma	‘*[Telehealth] allows some rural/regional patients to be managed* via *phone on some occasions, saving a visit into the clinic*’. Medical oncologist
	Consultation in the comfort of one's own home	‘*Able to be in my own home – a more relaxed atmosphere*’. Male, 72, advanced stage melanoma	*Sub‐theme did not emerge in clinician sample*
	Overcoming patient physical barriers	‘*At an advanced age, and with mobility issues … telehealth is a substantial complementary means of seeking/receiving advice*’. Male, 95, unknown stage	*Sub‐theme did not emerge in clinician sample*
	Ease of use	‘*It [telehealth] was convenient and easy. It only involved the telephone*’. Male, 77, early‐stage melanoma	‘*Easy for pt [patients]*’. Medical oncologist
Safety	Reduction of infection risk (COVID‐19 or otherwise)	‘*COVID was a risk as I travelled by public transport*’. *Male, 86, early‐stage melanoma*	‘*[Telehealth] allows at least some degree of assessment if patients (or doctors!) are not attending when they are unwell*’. Surgical oncologist
Improved access to care	Improved access to specialist care	‘*Living in a regional area, it gave me access to the top experts in Sydney…*’ Female, 78, advanced stage melanoma	*Sub‐theme did not emerge in clinician sample*
	Improved family support	‘*One big benefit for me was the presence of all my family members during my telehealth consultation*’. Male, 74, early‐stage melanoma	*Sub‐theme did not emerge in clinician sample*
	Maintaining regular contact	‘*[Telehealth] maintain[s] regular consults with my doctor*’. Female, 56, advanced stage melanoma	‘*Ability to maintain contact and provide continuity of care when face‐to‐face consultations are not easily possible or impossible*’. Surgical oncologist
	During COVID‐19 lockdowns	‘*…Under COVID‐19 type restrictions it enables me to have at least some preliminary contact/consultation with my melanoma health care professionals*’. Male, 76, advanced stage melanoma	‘*Happy with [telehealth] for quick follow‐up appointments mainly in patients who … cannot travel because of COVID‐19*’. Dermatologist
*Telehealth is appropriate under specific circumstances*			
Follow‐up		‘*My telehealth consultation was in follow‐up of melanoma surgery and saved a trip from … my home to Sydney*’. Male, 76, advanced stage melanoma	‘*It [telehealth] is great for follow‐up or triage of a lesion of concern*’. Dermatologist
Triage		‘*It was important to have information so that a plan could be put in place and face‐to‐face meetings organised based on urgency*’. Male, unknown age, advanced stage melanoma	‘*I consider the patient video just a triage tool*’. Dermatologist
When no physical exam is needed		‘*Where no physical is required, [telehealth allows] more efficient use of both the medical professional and my time*’. Male, 53, advanced stage melanoma	‘*[Telehealth is] impossible for real skin or melanoma checks*’. Dermatologist
Multidisciplinary discussions		*Theme did not emerge in patient sample*	‘*I think it [telehealth] should be used dr [doctor] to dr [doctor] [or] for GPs to send dermoscopy photos to the dermatologist using store‐and‐go instead of face‐to‐face*’. Dermatologist
Preparation		‘*Scans taken locally were used for my Dr to analyse my current status and offer diagnosis and procedures*’. Male, unknown age, early‐stage melanoma	‘*[Telehealth has] some value if patients email us photos of lesions they are concerned about*’. General practitioner
Decision to offer telehealth is based on patient characteristics		‘*Being a Stage IV cancer patient means I require more contact with my doctors and therefore influences whether telehealth applies or not*’*. Female, 27, advanced stage melanoma*	‘*Pt [patient] selection. Early during COVID a significant number of pts [patients] who were not well suited to telehealth were included. More recently smaller numbers are being seen, and pt [patients] are being selected based on the clinical situation*’. Medical oncologist
*How can telehealth be improved?*			
Technical	Add video	‘*My experience is only with phone calls so video calls may be preferential*’. Female, 56, Advanced stage melanoma	*Sub‐theme did not emerge in clinician sample*
	Virtual waiting rooms	*Sub‐theme did not emerge in patient sample*	‘*…dedicated program for telehealth on which patients could see a waiting room and their turn in the waiting room. Currently, if clinics are running late, patients need to be notified by phone that the telehealth is running late too*’. Dermatologist
	Integrate telehealth better with medical records	*Sub‐theme did not emerge in patient sample*	‘*Automatically attached to file and Telehealth consult notes. Accessible and streamlined EMR [Electronic Medical Record] process during consult*’. General practitioner
Personal	Creating supportive environment	‘*All doctors reinforcing that the space is safe, communicating effectively*’. Female, 27, advanced stage melanoma	*Sub‐theme did not emerge in clinician sample*
	Improve familiarity with technology	‘*Provide a better experience for older patients that do not know how to use the equipment or technology*’. Male, 66, early‐stage melanoma	‘*We will get used to it, but training for patients and doctors could help also*’. Medical oncologist
Clinical	Increase preparation	‘*Sharing reports of scans prior to appointment so I can prepare questions*’. Male, 46, advanced stage melanoma	‘*Good provision of information and photos by pt [patient] prior to consult*’. Surgical oncologist

#### 3.3.2 Advantages of telehealth

Both patients and clinicians identified convenience, safety and improved access to care as the primary advantages of telehealth (Table 2; Figure 4). Patients and clinicians indicated that telehealth was easy to use and had the potential to overcome geographical distance, especially for patients who lived outside metropolitan areas. Furthermore, patients noted time and cost savings because of reduced travel. Some patients noted the comfort of having telehealth consultations at their home, especially when physical limitations made face‐to‐face contact challenging. Patients and clinicians acknowledged the utility of telehealth in reducing the risk of infection during COVID‐19. They also noted the potential of telehealth to not only improve access to care during COVID‐19 lock down, but also maintain more regular follow‐up contact between clinicians and patients when needed. Furthermore, patients noted that telehealth improved access to specialist care, and improved the ability to have family involvement during consultations.

#### 3.3.3 Telehealth is helpful under specific circumstances

Patients and clinicians identified that telehealth could be useful to provide triage and routine follow‐up support when the melanoma disease is stable, and no physical examination is needed (Table 2; Figure 4). They commented that telehealth services could be helpful with preparation (e.g. taking scans locally or sending pictures for doctors to review). Clinicians thought telehealth could be appropriate for multidisciplinary discussions and reported typically making decisions to offer telehealth based on patient characteristics and needs.

#### 3.3.4 Telehealth improvements are required

Both patients and clinicians agreed that improved familiarity with the technology through experience or training would enhance the telehealth experience (Table 2; Figure 4). Also, they agreed that information sharing prior to the appointment would be beneficial. Additionally, patients thought that the addition of video to telephone consults and creating a supportive environment would improve their telehealth experience. Lastly, clinicians wanted to improve integration of telehealth with medical records and have a virtual waiting room available for patients, the latter being helpful when clinics are running behind schedule.

## 4 DISCUSSION

With increasing incidence and survival rates, it is expected that the number of Australians living with melanoma will double by 2030,^36^ leading to more patients needing routine follow‐up. Telehealth has the potential to increase access to melanoma care, especially for people living outside metropolitan areas, who may experience substantial barriers to accessing adequate services,^37^, ^38^ potentially leading to poorer clinical outcomes.^37^ COVID‐19 prompted rapid utilisation of telehealth,^39^, ^40^ which provided an opportunity to assess satisfaction with this mode of service delivery. This mixed methods study investigated satisfaction with follow‐up melanoma‐specific telehealth consultations from both patient and clinician perspectives.

While most patients reported that they received adequate care through telehealth, where their issues were appropriately addressed, only a minority reported that telehealth improved their care. This was echoed by the clinicians—most felt comfortable during telehealth consultations and believed that they delivered an adequate service, while still expressing a strong preference for face‐to‐face consultations. These views are consistent with those of other cancer patients^12^, ^15^, ^16^, ^18^, ^20^, ^21^, ^22^, ^41^ and their clinicians,^14^, ^15^, ^18^, ^41^ which suggests that telehealth cannot replace face‐to‐face consultations.

Both patients and clinicians have identified the inability to conduct physical examinations via telehealth to be a major shortcoming, consistent with previous studies in other cancers.^14^, ^15^, ^18^, ^19^, ^23^, ^40^ Physical examination is an essential part of melanoma follow‐up,^42^ as it adds an objective assessment to any concerns identified by a patient, often increasing patient confidence in the clinical opinion, in addition to providing opportunistic screening for other health conditions.^19^ Patient‐assisted virtual physical examinations may provide a substitute for physical examinations by clinicians via telehealth, and some guidelines are emerging for general healthcare.^43^

Patients and clinicians identified telehealth as having several technical and interpersonal shortcomings. Both groups shared the concern that they struggled with having access to adequate technology and feeling comfortable using it. Rapid transition to telehealth resulting from COVID‐19^44^ may not have allowed sufficient time to source adequate technology and provide appropriate training for both clinicians and patients, which may also explain why the majority of telehealth consultations in this study were conducted via telephone rather than audio‐visually. Additionally, both patients and clinicians perceived telehealth communication to be less personal and more difficult due to reduced availability of non‐verbal cues, consistent with the findings from other cancer groups.^12^, ^14^, ^23^, ^39^, ^41^ Good interpersonal communication and rapport are especially important when symptoms and emotional issues are discussed,^21^, ^40^ and have been shown to be improved by the inclusion of an audio‐visual medium.^19^, ^41^

Clinicians and patients were congruent in expressing convenience, safety and improved access to care as benefits of telehealth, consistent with previously reported research.^18^, ^20^, ^23^, ^41^ Patients also reported that telehealth allowed family and friends to be present in a supportive role during the consultation, without a major impact on the family member/friend's routines. This is in contrast with previous research in cancer patients, which identified that family and friends in supportive roles were less likely to attend telehealth appointment due to scheduling difficulties and patients not being aware of the opportunity.^12^ This highlights the importance of explicitly encouraging patients to include support people in telehealth appointments, consistent with best practice.^45^, ^46^

Notwithstanding the disadvantages, convenience and improved access to timely and specialist care are the benefits of telehealth that have the greatest potential to extend outside the COVID‐19 context, especially for patients who are socially and geographically isolated. Both patients and clinicians in the present study could see the potential of telehealth as a vital part of wholistic melanoma care. A hybrid telehealth model has been proposed by Burbury and colleagues,^13^ where telehealth could be offered in conjunction with face‐to‐face care, and in consultation with patients. When needed, physical examination could be conducted in collaboration with a local healthcare provider.^13^ Adding to these considerations, the present study indicates that telehealth may be appropriate for triage and follow‐up consultations when a patient's disease is stable. Furthermore, clinicians thought that telehealth may be beneficial for multidisciplinary consultations. In our sample, the importance of the consideration of patient and clinical factors was also highlighted. Our results indicate that, in comparison to people diagnosed with early melanoma, those diagnosed with advanced disease were more likely to be satisfied with telehealth. This may be a result of a relatively higher frequency of follow‐up in patients diagnosed with advanced disease,^3^ which may allow for more opportunities to develop rapport and relationship. Our study also found some evidence that patients who perceived difficulties in their communication and relationship with their oncologist tend to have lower satisfaction with telehealth. Additionally, those who reported higher supportive care concerns and who were not experienced in using telehealth tended to have lower telehealth satisfaction. If telehealth is offered to these patients, their clinicians may wish to discuss additional guidance and support strategies with them, to ensure both have a satisfactory experience. Guidelines,^43^ as discussed above, may offer a starting point for these discussions.

If telehealth is adopted as a part of routine melanoma care, several enhancements could be made to improve satisfaction. Providing consultations audio‐visually may improve communication between patient and clinician. Providing video telehealth consultations is also preferred by the Australian government, with the provision of permanent MBS items which prioritise video telehealth consultations.^47^ Organisational support is needed to invest in the most appropriate videoconferencing platforms, with suitable platforms already documented.^13^ Virtual waiting rooms may be beneficial in communicating with patients when a clinician is late.^20^ Closed captioning may be beneficial for people who are hard of hearing.^48^ Lastly, training and support may increase comfort with telehealth, as indicated by some of our participants. Specific advice and guidance for patients and clinicians is available.^13^ Training programs, both integrated into university curricula and provided as part of continuing professional development, have been shown to improve clinician competence with telehealth delivery.^49^

This study has several limitations. First, our recruitment rates were lower than anticipated, similar to experiences of other researchers during COVID‐19.^50^ This may have led to potential selection bias, where participants interested in telehealth were more likely to respond. We tried to manage this by offering study materials electronically and in paper format, so that people less familiar with technology had an opportunity to participate. Our sample's sex and age were consistent with Australian melanoma population,^51^ with participants' reported educational and income levels similar to general Australian population.^52^, ^53^ Second, we asked participants to reflect on their most recent telehealth consultation, which may have occurred sometime prior to data collection, leading to potential recall bias. Third, we collected data from three sites, all located in metropolitan Sydney, which may limit generalisation of results to all melanoma patients. Furthermore, the small clinician sample size was not sufficient to investigate potential factors associated with clinician satisfaction with telehealth and may limit generalisability. Nevertheless, we found clinician views to be consistent with both the patients' reports and previously published research in other cancers, which increases the confidence in our results. Lastly, overwhelming majority of consultations were delivered via telephone, which may limit our findings to this mode of telehealth delivery.

Our study has several strengths. We used validated questionnaires to measure satisfaction with telehealth and supplemented formal scales with additional questions based on previous research. Using mixed methods, we balanced quantifying important variables and allowed participants to provide rich open‐ended responses, which has provided context for the quantitative data. Lastly, patient sample size was sufficient for all planned analyses.

Future studies should validate our results in a larger, representative melanoma population. While we were able to identify some groups of patients who may be more likely to experience lower satisfaction with telehealth, this needs replication and mechanisms for these relationships should be investigated in order to be able to provide effective support. Furthermore, scales measuring satisfaction with telehealth should be fine‐tuned and validated further as there were several areas that investigators felt were not covered, resulting in using additional questions from Becevic et al.^26^ Further, having a similar response format for all HOTAQ questions would allow calculation of the overall satisfaction score and improve reliability. Additionally, investigation of telehealth models for melanoma follow‐up would be beneficial. This is especially important given the recent announcement of an Australian Melanoma Nurse Program, which will expand telehealth nursing services (planned to be implemented in 2025–2026). Its aim is to provide information, care and support to melanoma patients.

In conclusion, telehealth may offer another avenue for service delivery that may complement face‐to‐face consultations and may be especially helpful to people with limited access to melanoma care. Organisational, policy and funding support is needed to ensure that technology and training are provided so that patients and clinicians find the encounter efficient and satisfactory.

## AUTHOR CONTRIBUTIONS

**Ali Al‐Rikaby:** Conceptualization (supporting); data curation (equal); formal analysis (equal); investigation (equal); methodology (equal); project administration (lead); writing – original draft (equal); writing – review and editing (equal). **Ahmad Sulaiman:** Formal analysis (supporting); writing – original draft (supporting); writing – review and editing (supporting). **Jake R. Thompson:** Conceptualization (supporting); data curation (supporting); formal analysis (supporting); investigation (supporting); methodology (supporting); project administration (supporting); software (lead); supervision (supporting); visualization (lead); writing – original draft (equal); writing – review and editing (equal). **Robyn P. M. Saw:** Conceptualization (equal); data curation (equal); investigation (equal); methodology (equal); supervision (lead); writing – original draft (equal); writing – review and editing (equal). **Frances Boyle:** Conceptualization (equal); investigation (equal); methodology (equal); writing – original draft (equal); writing – review and editing (equal). **Nicole Taylor:** Conceptualization (equal); data curation (equal); investigation (equal); project administration (equal); writing – original draft (equal); writing – review and editing (equal). **Matteo S. Carlino:** Conceptualization (equal); data curation (equal); investigation (equal); methodology (equal); writing – original draft (equal); writing – review and editing (equal). **Rachael L. Morton:** Conceptualization (equal); investigation (equal); methodology (equal); writing – original draft (equal); writing – review and editing (equal). **Omgo E. Nieweg:** Conceptualization (equal); data curation (equal); investigation (equal); methodology (equal); writing – original draft (equal); writing – review and editing (equal). **John F. Thompson:** Conceptualization (equal); data curation (equal); investigation (equal); methodology (equal); project administration (equal); writing – original draft (equal); writing – review and editing (equal). **Iris Bartula:** Conceptualization (lead); data curation (equal); formal analysis (equal); funding acquisition (lead); investigation (lead); methodology (lead); project administration (supporting); supervision (lead); writing – original draft (equal); writing – review and editing (equal).

## FUNDING INFORMATION

This work and researchers' time was supported by Melanoma Institute Australia and the Bill and Patricia Ritchie Foundation. IB and FB are supported but the Friends of the Mater Foundation. RLM was supported by a University of Sydney, Robinson Fellowship and an NHMRC Investigator grant #1194703. This work formed part of the requirements for a Bachelor of Medical Science Honours degree (University of Sydney) for AAR, under the supervision of RPMS and IB.

## CONFLICT OF INTEREST STATEMENT

RPMS has received honoraria for advisory board participation from MSD, Novartis and Qbiotics and speaking honoraria from BMS and Novartis. JFT has received honoraria for advisory board participation from BMS Australia, MSD Australia, GSK and Provectus Inc, and travel and conference support from GSK, Provectus Inc and Novartis. AS, JRT, FB and IB report no conflicts of interest.

## ETHICS STATEMENTs

Ethical approval was granted by Sydney Local Health District—Royal Prince Alfred Zone (2020/ETH02519). Participants provided written informed consent.

## Supporting information


Table S1.

## Data Availability

Following publication, data will be available for sharing to methodologically—sound, Human Research Ethics Committee—approved studies. Interested investigators are encouraged to contact corresponding author with proposals.
