# Supplementary material for: Telehealth follow‐up consultations for melanoma patients during the COVID‐19 pandemic: Patient and clinician satisfaction
Source: Cancer Med. 2023 Nov 6;12(23):21373–88. doi: 10.1002/cam4.6679 (PMC10726917; doi:10.1002/cam4.6679)
Supplement: Supplementary file 1 — Table S1. [file CAM4-12-21373-s001.docx]

# Supplementary Material

Supplementary Table 1. Associations of Demographic, Clinical and Psychosocial Variables with Telehealth Satisfaction

| **Pearson Correlation** | | | | | | | | | | | | |
| --- | --- | --- | --- | --- | --- | --- | --- | --- | --- | --- | --- | --- |
| **Variable** | | | n | | r | | p | |  |  |  |  |
| Fear of cancer recurrence | | | 98 | | -0.02 | | 0.82 | |  |  |  |  |
| **Spearman’s Rank Correlations** | | | | | | | | | | | | |
| **Variable** | | | n | | r | | p | |  |  |  |  |
| Age | | | 90 | | 0.10 | | 0.33 | |  |  |  |  |
| Trust in oncologist | | | 92 | | 0.28 | | **0.01*** | |  |  |  |  |
| Global Health Status | | | 98 | | 0.10 | | 0.34 | |  |  |  |  |
| Treatment concerns and future disease risk | | | 96 | | 0.12 | | 0.23 | |  |  |  |  |
| Disease prognosis and acceptance | | | 96 | | -0.04 | | 0.69 | |  |  |  |  |
| Care delivery and communication | | | 93 | | -0.29 | | **0.01*** | |  |  |  |  |
| Supportive care | | | 92 | | -0.29 | | **0.01*** | |  |  |  |  |
| **Independent-Samples T Tests** | | | | | | | | | | | | |
| **Variable** | n | $\mu$ | | SD | | df | | t | p | | | d |
| Sex |  |  | |  | |  | |  |  | | |  |
| Male | 53 | 76.55 | | 13.05 | |  | |  |  | | |  |
| Female | 45 | 74.80 | | 10.99 | | 96 | | 0.71 | 0.49 | | | 0.15 |
| Residence |  |  | |  | |  | |  |  | | |  |
| Metropolitan | 60 | 75.67 | | 12.23 | |  | |  |  | | |  |
| Rural/Remote | 36 | 75.69 | | 12.06 | | 94 | | 0.01 | 0.99 | | | 0.00 |
| Living Arrangements |  |  | |  | |  | |  |  | | |  |
| Living Alone | 15 | 75.80 | | 12.80 | |  | |  |  | | |  |
| Living with Someone | 83 | 75.72 | | 12.07 | | 96 | | 0.02 | 0.98 | | | 0.01 |
| Melanoma Stage |  |  | |  | |  | |  |  | | |  |
| Early (Stage 0-II) | 45 | 72.84 | | 10.77 | |  | |  |  | | |  |
| Advanced (Stage III-IV) | 44 | 79.75 | | 11.87 | | 87 | | 2.88 | **0.01*** | | | 0.61 |
| **Mann-Whitney U Test** | | | | | | | | | | | | |
| **Variable** | n | Mean rank | | Sum of ranks | | U | | Z | p | | |  |
| Clinical Trial |  |  | |  | |  | |  |  | | |  |
| Yes | 15 | 45.77 | | 686.50 | |  | |  |  | | |  |
| No | 77 | 46.64 | | 3591.50 | | 566.50 | | -0.12 | 0.91 | | |  |
| **Analysis of Variance (ANOVA) Tests** | | | | | | | | | | | | |
| **Variable** | n | $\mu$ | | SD | | df | | F | p | | | $\eta$^2^ |
| Employment Status |  |  | |  | |  | |  |  | | |  |
| Employed | 28 | 73.29 | | 8.98 | |  | |  |  | | |  |
| Other | 5 | 81.00 | | 10.03 | |  | |  |  | | |  |
| Retired | 65 | 76.38 | | 13.31 | | 2, 95 | | 1.14 | 0.32 | | | 0.02 |
| Educational Attainment |  |  | |  | |  | |  |  | | |  |
| Secondary | 42 | 75.21 | | 12.61 | |  | |  |  | | |  |
| Tertiary | 34 | 75.76 | | 10.82 | |  | |  |  | | |  |
| Vocational | 22 | 76.68 | | 13.52 | | 2, 95 | | 0.10 | 0.90 | | | 0.00 |
| Annual Income |  |  | |  | |  | |  |  | | |  |
| <$62,000 AUD | 61 | 77.82 | | 13.47 | |  | |  |  | | |  |
| $62,000-$121,999 AUD | 10 | 73.30 | | 6.78 | |  | |  |  | | |  |
| ≥$120,000 AUD | 7 | 71.57 | | 10.94 | | 2, 75 | | 1.17 | 0.32 | | | 0.03 |
| Time Since Diagnosis |  |  | |  | |  | |  |  | | |  |
| <12 Months | 14 | 74.36 | | 10.16 | |  | |  |  | | |  |
| 13-23 Months | 13 | 70.85 | | 12.84 | |  | |  |  | | |  |
| 24 Months | 68 | 75.38 | | 12.03 | | 2, 92 | | 1.236 | 0.30 | | | 0.03 |
|  |  |  | |  | |  | |  |  | | |  |
|  |  |  | |  | |  | |  |  | | |  |
| **Analysis of Variance (ANOVA) Tests (continued)** | | | | | | | | | | | | |
| **Variable** | n | $\mu$ | | SD | | df | | F | p | | | $\eta$^2^ |
| Previous Telehealth Usage |  |  | |  | |  | |  |  | | |  |
| 1 | 41 | 71.39 | | 13.09 | |  | |  |  | | |  |
| 2-5 | 44 | 78 | | 9.67 | |  | |  |  | | |  |
| >5 | 12 | 83 | | 12.34 | | 2, 94 | | 6.119 | **<0.01*** | | | 0.12 |

**Bold* -** statistically significant at α=0.05; df - degrees of freedom; SD - standard deviation.
